# Proteomic analyses reveal new features of the box H/ACA RNP biogenesis

**DOI:** 10.1093/nar/gkad129

**Published:** 2023-03-03

**Authors:** Florence Schlotter, Salim Mérouani, Justine Flayac, Valentyne Kogey, Amani Issa, Maxime Dodré, Alexandra Huttin, Christiane Branlant, Edouard Bertrand, Stéphane Labialle, Franck Vandermoere, Céline Verheggen, Séverine Massenet

**Affiliations:** Université de Lorraine, CNRS, IMoPA, F-54000 Nancy, France; Université de Lorraine, CNRS, IMoPA, F-54000 Nancy, France; Université de Lorraine, CNRS, IMoPA, F-54000 Nancy, France; Université de Lorraine, CNRS, IMoPA, F-54000 Nancy, France; Université de Lorraine, CNRS, IMoPA, F-54000 Nancy, France; Université de Lorraine, CNRS, IMoPA, F-54000 Nancy, France; Université de Lorraine, CNRS, IMoPA, F-54000 Nancy, France; Université de Lorraine, CNRS, IMoPA, F-54000 Nancy, France; IGH, CNRS, Univ Montpellier, Montpellier, France; Université de Lorraine, CNRS, IMoPA, F-54000 Nancy, France; IGF, CNRS, INSERM, Univ Montpellier, Montpellier, France; IGH, CNRS, Univ Montpellier, Montpellier, France; Université de Lorraine, CNRS, IMoPA, F-54000 Nancy, France

## Abstract

The conserved H/ACA RNPs consist of one H/ACA RNA and 4 core proteins: dyskerin, NHP2, NOP10, and GAR1. Its assembly requires several assembly factors. A pre-particle containing the nascent RNAs, dyskerin, NOP10, NHP2 and NAF1 is assembled co-transcriptionally. NAF1 is later replaced by GAR1 to form mature RNPs. In this study, we explore the mechanism leading to the assembly of H/ACA RNPs. We performed the analysis of GAR1, NHP2, SHQ1 and NAF1 proteomes by quantitative SILAC proteomic, and analyzed purified complexes containing these proteins by sedimentation on glycerol gradient. We propose the formation of several distinct intermediate complexes during H/ACA RNP assembly, notably the formation of early protein-only complexes containing at least the core proteins dyskerin, NOP10, and NHP2, and the assembly factors SHQ1 and NAF1. We also identified new proteins associated with GAR1, NHP2, SHQ1 and NAF1, which can be important for box H/ACA assembly or function. Moreover, even though GAR1 is regulated by methylations, the nature, localization, and functions of these methylations are not well known. Our MS analysis of purified GAR1 revealed new sites of arginine methylations. Additionally, we showed that unmethylated GAR1 is correctly incorporated in H/ACA RNPs, even though with less efficiency than methylated ones.

## INTRODUCTION

Many cellular functions are achieved by a family of molecular machines made of RNA-protein complexes, called non-coding ribonucleoprotein particles (ncRNP). These ncRNPs include ribosomes and spliceosomes, respectively involved in translation and pre-mRNA splicing, and a plethora of other stable ncRNPs involved in multiple cellular functions. Defects in ncRNP assembly lead to severe human pathologies (1–4). For the cells, it is then essential to produce these ncRNPs in a functional state. The biogenesis of ncRNPs represents fascinatingly intricate processes. It involves multiple steps that have to be coordinated and successive assemblies of large multiprotein pre-RNP complexes. Despite the tendency for self-assembly of ncRNPs *in vitro*, the situation *in vivo* is extremely different and numerous assembly factors are required for efficient and faithful assembly in cells. These so-called assembly factors intervene transiently and fulfill various functions: they chaperone the RNP constituents, promote the formation of the pre-RNP complexes and control their quality, increase the specificity of assembly, transport subunits, etc (3,5–8).

The box H/ACA family of ncRNPs are conserved in archaea and eukaryotes and catalyze the site-specific conversion of uridine (U) into pseudouridine (Ψ) of target RNAs (5,9,10). Ψ residues are the most frequent post-transcriptional modifications on RNAs. They stabilize RNA structure by improving base stacking and allowing the formation of an additional hydrogen bond. Over the years, it was shown that Ψs in RNAs improve greatly RNA functions. For instance, Ψs in ribosomal RNAs (rRNAs) and U-rich small nuclear RNAs (UsnRNAs) play important roles in protein translation and splicing, respectively (11–13). In eukaryotes, small nucleolar box H/ACA RNPs (snoRNPs) localize in the nucleolus where they post-transcriptionally modify rRNAs and U6 snRNA (5,9,10,12). A few of them are also required for pre-rRNA cleavage steps. Besides canonical H/ACA snoRNPs, related families are also present in cells. Small Cajal body-specific H/ACA RNPs (scaRNPs) structurally resemble H/ACA snoRNPs but they are retained in the Cajal Bodies (CBs) where they catalyze Ψ formation in UsnRNAs other than U6. Vertebrate telomerase RNA that synthesizes telomeric DNA also contains a H/ACA motif and localizes in CBs (14,15). Another family of H/ACA RNAs of unknown function is processed for intronic Alu repetitive sequences (Alu ACA RNAs) (16). The full repertoire of box H/ACA RNPs may likely be larger than anticipated with multiple crucial functions in cells. Many components and assembly factors of H/ACA RNPs are mutated in the inherited bone marrow failure syndrome dyskeratosis congenita (DC) (12,15). As a consequence, critically short telomeres are a hallmark of all DC patients.

Box H/ACA RNAs share similar hairpin-hinge-hairpin-tail secondary structures. The single-stranded hinge region contains the conserved H box. Another conserved sequence, the ACA box, is located in the tail, three nucleotides upstream from the 3’ termini of the RNAs. Each hairpin is associated with a heterotetramer composed of GAR1, NHP2, NOP10, and dyskerin proteins (CBF5, NAP57) (5,9,10,12). The latest catalyzes the modification. CryoEM analysis of purified mammalian telomerase enzyme revealed that the organization of the two heterotetramers is asymmetric and that they interact with each other by the formation of a dyskerin/dyskerin dimer (17,18). In the RNP, NOP10 and GAR1 bind independently to two orthogonal faces of the dyskerin catalytic domain (17–22). All proteins are required for optimal enzymatic activity and cell survival. Each hairpin presents an internal loop called the pseudourydilation pocket which contains a short sequence complementary to the RNA target. This RNA target is recruited through base-pairing with the H/ACA RNA guide and extensive interactions with proteins. NHP2 belongs to the L7Ae family of RNA-binding proteins. Eukaryotic NHP2 associates through a protein-protein interaction with NOP10 but has a poor RNA-binding specificity (22,23). Even though NHP2 does not bind to box H/ACA RNAs or substrate RNAs, it seems to be critical for pseudouridylation by allowing the correct positioning of the target U in the catalytic site (24). Both the archaeal and eukaryotic RNPs require GAR1 for substrate loading, catalysis, and turnover during the enzymatic reaction (22,25–27). GAR1 does not contact permanently box H/ACA RNAs or substrate RNAs (25,26,28) but its transient interaction with substrate RNAs can promote their release from H/ACA particles (25).

Catalytically active archaeal and yeast H/ACA RNPs can be reconstituted using *in vitro* transcribed RNA and recombinant proteins (22,24,29,30). However, the *in vivo* assembly of eukaryotic H/ACA RNPs requires several assembly factors, including the essential and conserved NAF1 and SHQ1 proteins that seem to be absent in archaea. These assembly factors associate transiently with H/ACA pre-RNPs during their biogenesis but are not integral components of mature H/ACA particles. Depletion of NAF1 or SHQ1 leads to the destabilization of box H/ACA RNAs and pre-rRNA processing defects (31–34). SHQ1 seems to interact with dyskerin in the cytoplasm as soon as it is synthesized, thereby protecting it from aggregation and degradation, and preventing dyskerin from illicit binding to RNAs (22,35,36). In the nucleus, SHQ1 is released from H/ACA pre-RNPs with the help of the R2TP complex composed of the AAA + ATPases RUVBL1 and RUVBL2, associated with their co-factors RPAP3 and PIH1D1 (37,38). Another assembly factor NAF1, which interacts with the CTD of RNA polymerase II, recruits the core proteins dyskerin/NHP2/NOP10 to nascent H/ACA RNAs at their site of transcription (33,39,40). GAR1 is associated later in the maturation process, via a remodeling event that exchanges NAF1 for GAR1 probably in the CBs (40,41). NAF1-containing pre-RNPs are inactive and the replacement of NAF1 by GAR1 allows the formation of an active enzyme (22).

Besides NAF1 and SHQ1, other factors may intervene in H/ACA RNP biogenesis. The assembly factor NUFIP, which is involved in box C/D RNP and UsnRNP biogenesis, interacts directly with NHP2; and its depletion leads to reduced levels of box H/ACA RNAs (42). Its role in the H/ACA assembly still needs to be defined. The retention of H/ACA scaRNPs and telomerase in CBs requires TCAB1 (WDR79) that specifically interacts with the CAB sequence present within these RNAs (43–46). Nopp140 is also important to recruit and retain H/ACA scaRNPs in CBs (47) and is believed to function in H/ACA snoRNP traffic from CBs to nucleoli (48,49). Other factors are for a long time thought to participate in H/ACA RNP biogenesis but their roles are still enigmatic. The Survival of Motor Neurons complex (SMN) is one of these factors. The SMN complex is composed of the core SMN protein, associated with Gemin2-8 and Unrip proteins (3,50,51). This complex is localized throughout the cytoplasm and in nuclear bodies called gems, which correspond in most cells to CBs (52,53). The SMN complex is essential in cells and functions in multiple cellular pathways related to RNA metabolism, including transcription, splicing, RNP assemblies such as U snRNPs and Signal Recognition Particles, and mRNA transport in neurons (3,8,50,54,55). Deletions or mutations in the Survival of Motor Neurons (*SMN1*) gene lead to reduced SMN protein levels and cause Spinal Muscular Atrophy (SMA), a neuromuscular disease (56,57). It has been suggested early on that the SMN complex is involved in H/ACA RNP assembly because of the direct interaction between SMN and GAR1 and its association in cells with H/ACA RNPs including telomerase (58–60). Decreased levels of rRNA pseudouridylations and increased association of the H/ACA RNAs with NAF1 occur in mammalian cells depleted of both Unrip (one component of the SMN complex) and Nopp140 (61). Moreover, SMA patient cells show decreased localization of GAR1, dyskerin, and Nopp140 to CBs, indicating that reduced level of the SMN protein leads to disturbed localization of H/ACA core proteins and biogenesis factors (62). NUFIP and the SMN complex are associated in cells (63), suggesting functional links between these two assembly factors.

In this study, we used quantitative proteomics to identify the panel of proteins associated with two H/ACA core proteins, NHP2 and GAR1, and two assembly factors, NAF1 and SHQ1. We revealed new proteins that can be important for box H/ACA RNP assembly and/or functions. Moreover, biochemical assays allowed us to propose the formation of several distinct intermediate complexes during H/ACA RNP assembly. We showed that GAR1 associates with PRMT1 and the PRMT5/RIOK1/MEP50 complex and mapped the arginine methylation sites of purified GAR1 protein by mass spectrometry (MS).

## MATERIALS AND METHODS

### Cell culture, siRNAs, and DNA manipulations

Cells were maintained in DMEM supplemented with 10% of fetal bovine serum, penicillin/streptomycin (10 U/ml) and glutamin (2,9 mg/ml), in a humidified CO_2_ incubator at 37°C. HEK293 stable cell lines expressing NAF1, NHP2, SHQ1 or GAR1 with either Flag or GFP tags under the control of a tetracycline-regulated CMV/TetO_2_ promoter were created with the Flp-in T-REx system (Thermo Fisher Scientific), as recommended by the manufacturer, using the HEK293 Flp-in T-REx cell line and the pcDNA5/FR/TO plasmids encoding the desired ORFs and constructed by PCR cloning. Clones were selected in hygromycin B (100 μg/ml), picked individually, and characterized by WB.

### Antibodies

The following antibodies were used: anti-NAF1 (Abcam, ab122323) rabbit polyclonal, anti-SHQ1 (27020–1-AP) (Proteintech) rabbit polyclonal, anti-NHP2 (15128–1-AP) (Proteintech) rabbit polyclonal, anti-dyskerin (Santa Cruz H-300) rabbit polyclonal, anti-NOP10 (Abcam, ab134902) rabbit monoclonal, anti-GAR1 (11711–1-AP) (Proteintech) rabbit polyclonal, anti-GAPDH (Abcam, ab9485) rabbit polyclonal, anti-coilin (Bethyl, A303-759A) rabbit polyclonal, anti-nucleolin (Abcam, ab136649) mouse monoclonal, anti-S6 (ThermoS, 9H8L2) rabbit monoclonal, anti-L5 (Bethyl, A303-933A) rabbit polyclonal, anti-U1A (ThermoS, PA5-27474) rabbit polyclonal, and anti-fibrillarin (Abcam, ab166630) rabbit monoclonal.

### Co-immunoprecipitation and western blot (WB)

When required, the expression of the tagged proteins was induced for 3 or 12 h using 1 μg/ml of Doxycycline. When indicated, the cells were treated for 12 or 24 h with 20 μM ADOX before extract preparation. Total HEK293 Flp-In T-REx cell extracts were prepared as previously described (64) in RSB150 buffer (10 mM Tris–HCl pH 7.5, 150 mM NaCl, 2.5 mM MgCl_2_) containing 0,05% NP-40, and incubated with either (i) specific antibodies bound to protein A-Sepharose (Amersham), or (ii) with anti-Flag M2 affinity gel (Sigma-Aldrich), or (iii) with GFP-Trap (Chromotech) for 2 h at 4°C. When indicated, RNase A was added at 100 μg/ml. The beads were washed with the same buffer and the immunoprecipitated proteins were eluted in Laemmli buffer and analyzed by SDS-PAGE and WB using appropriate antibodies and ECL revelation kit (Ozyme). Systematically, the membranes were cut into pieces according to the molecular weight ladder loaded in parallel to the samples, to allow the probing by WB of multiple proteins for each IP experiment. When required, membranes were stripped according to the manufacturer protocol (Millipore) and probed a second time with other antibodies. Images were acquired and quantification was performed with Fusion Solo (Vilber). Kolmogorov–Smirnov or Wilcoxon tests were calculated from four to six independent experiments using GraphPad Prism.

### Purification and analysis of H/ACA complexes on glycerol gradients

IPs were performed as described above using anti-Flag M2 affinity gel (Sigma-Aldrich), and total cellular extracts prepared from 10 × 10 cm plates in 500 μl of RSB 150 buffer containing 0,05% NP-40 from HEK293 Flp-In T-REx cells expressing Flag-NHP2 or Flag-NAF1, in the absence or the presence of RNAse A (100 μg/ml). The beads were washed, and the complexes containing the Flag-tagged protein were eluted from the beads by incubation for 1h with 500 μg/ml of Flag Peptide (Sigma-Aldrich). Purified H/ACA complexes pooled from four IPs were concentrated using Amicon ultra 3K (Millipore). Purified concentrated complexes or total cellular extracts were loaded on a linear 10–30% glycerol gradient prepared in the same buffer using a Gradient Master (Serlabo), and fractionated by centrifugation through the gradients in an SW41 rotor at 4°C for 17 h at 25 000 rpm using the Optima XE-90 ultracentrifuge (Beckman). Eighteen fractions were collected from the top of the gradients with a FoxyR1 (Teledyne) density gradient fractionation system. Each fraction was concentrated using Amicon ultra 3K (Millipore), and 1/3 (for the total extract) or the total (for the purified complexes) of each fraction was analyzed by SDS-PAGE and WB.

### Immunofluorescence staining (IF) and image acquisition

HEK293 Flp-In T-REx expressing GFP-NHP2 cells were plated at 120000 cells/well (6-well plates) onto glass coverslips for 48 h. The expression of GFP-NHP2 was induced either 3 or 12 h before the IF experiment. Induced cells were fixed in 2% paraformaldehyde/PBS 1× for 10 min at RT. After three washes with PBS 1×, cells were permeabilized in 0.5% Triton X-100 for 10 min at RT, then rinsed 3 times with PBS 1X. Blocking was performed for 30 min at 4°C in PBS 1× containing 3% bovine serum albumin (BSA). Coverslips were then incubated for 2 h at 4°C with a mix of two primary antibodies diluted in PBS 1X: anti-coilin at 1:400 (Bethyl, A303-759A) and anti-nucleolin at 1:200 (Abcam, ab136649). After rinsing in PBS 1×, coverslips were then incubated for 30 min at RT with a mix of secondary antibodies coupled to Alexa fluorophore dyes 633 nm (Invitrogen A21053) and 555 nm (Invitrogen A21428), diluted in PBS 1× at 1:200. After three washes with PBS 1× and a quick dry, coverslips were mounted on a slide with a mounting medium supplemented with DAPI to counterstain nuclei (Duolink In Situ Mounting Medium with DAPI – Sigma DUO82040). Images were acquired by a laser confocal microscope Leica SP5-X. Images from each channel were recorded separately and then merged. Image analysis and processing were performed with the ImageJ software.

### SILAC IP and proteomic analysis

SILAC experiments were performed as previously described (65). Six 15-cm diameter plates per condition of HEK293 Flp-In T-REx cells inducibly expressing the GFP-tagged proteins were grown for 15 days in each isotopically labeled media (CIL/Eurisotop), to ensure complete incorporation of isotopically l-lysine–^2^HCl/l-arginine–HCl light label (K0R0 or L condition, corresponding to the control), l-lysine–^2^HCl (^2^H4, 96–98%)/l-arginine–HCl (^13^C6, 99%) semi-heavy label (K4R6 or M condition), or l-lysine–^2^HCl (^13^C6, 99%; ^15^N2, 99%)/l-arginine–HCl (^13^C6, 99%; ^15^N4, 99%) heavy label (K8R10 or H condition) (percentages represent the isotopic purity of the labeled amino acids). Control HEK293 Flp-In T-REx cells that do not express the GFP fusion were cultured in K0R0 SILAC condition, while Flp-In cells expressing GFP-NHP2, GFP-SHQ1, and GFP-NAF1 were cultured in K4R6, and Flp-In cells expressing GFP-GAR1 in K8R10. After 3h of induction of the expression of the GFP-tagged protein by adding 1 μg/ml Doxycyclin in the culture media, cells were rinsed with PBS, trypsinized, and cryogrinded. The powder was resuspended in HNT lysis buffer (20 mM HEPES, pH 7.4, 150 mM NaCl, 0.5% Triton X-100, protease inhibitor cocktail (cOmplete, Roche)). Extracts were incubated for 20 min at 4 °C and clarified by centrifugation for 10 min at 20 000 g. For all IP experiments, extracts were pre-cleared by incubation with Protein G Sepharose beads (GE Healthcare) for 1 h at 4 °C. Each extract was then incubated with 50 μl of GFP-Trap beads (Chromotek) for 1.5 h at 4°C, washed five times with HNT buffer, and beads from the different isotopic conditions were finally pooled. Bound proteins were eluted by adding 1% SDS to the beads and boiling them for 10 min. Proteomic analysis was performed as previously described (65). Enrichment is calculated as a SILAC ratio M/L or H/L. SILAC IP of NHP2 and GAR1 were done concomitantly in a differential analysis (M condition for NHP2 and H condition for GAR1). SILAC IP of NAF1 and SHQ1 were done using M condition.

### GAR1 arginine methylation analysis by mass spectrometry

The expression of GFP-GAR1 in HEK293 Flp-In T-REx 293 cells was induced for 12 h by 1 μg/ml of Doxycycline. Total extracts were prepared as previously described (64) in RSB 600 buffer (10 mM Tris–HCl, pH 7.5, 600 mM NaCl, 2.5 mM MgCl_2_) containing 1% NP-40 and incubated with GFP-Trap beads (Chromotech) for 2 h at 4°C. The beads were washed first with the same buffer, then with RSB 150 buffer (10 mM Tris–HCl, pH 7.5, 150 mM NaCl, 2.5 mM MgCl_2_) containing 0,05% NP-40. The immunoprecipitated material was resuspended in Laemmli buffer and separated by SDS-PAGE. After colloidal blue staining of the gel, the band corresponding to GFP-GAR1 molecular weight was cut and digested with chymotrypsin (10 ng/ml, O/N). Digested peptides were analyzed by LC–MSMS using the method previously described in ‘SILAC IP’ experiment. Peak lists obtained from MS/MS spectra were identified using X!Tandem version Vengeance (2015.12.15.2) (66). The search was conducted using SearchGUI version 3.2.2.0 (67). Protein identification was conducted against a concatenated target/decoy (68) version of the Homo sapiens complement of the UniProtKB (69) (reviewed and isoforms version, downloaded on 11-19-2020, 96456 (target) sequences). The decoy sequences were created by reversing the target sequences in SearchGUI. The identification settings were as follows: Chymotrypsin, Specific, with a maximum of 2 missed cleavages 10.0 ppm as MS1 and 0.02 Da as MS2 tolerances; fixed modifications: Carbamidomethylation of C (+57.021464 Da), variable modifications: Oxidation of M (+15.994915 Da), Dimethylation of K (+28.0313 Da), Dimethylation of R (+28.0313 Da), Methylation of K (+14.01565 Da), Methylation of R (+14.01565 Da), Trimethylation of K (+42.04695 Da), fixed modifications during refinement procedure: Carbamidomethylation of C (+57.021464 Da), variable modifications during refinement procedure: Acetylation of protein N-term (+42.010565 Da), Pyrolidone from E (–18.010565 Da), Pyrolidone from Q (–17.026549 Da), Pyrolidone from carbamidomethylated C (–17.026549 Da). Peptides and proteins were inferred from the spectrum identification results using PeptideShaker version 1.16.44 (70). Peptide Spectrum Matches (PSMs), peptides and proteins were validated at a 1.0% False Discovery Rate (FDR) estimated using the decoy hit distribution. Post-translational modification localizations were scored using the d-score (71) and the phosphoRS score (72) with a threshold of 95.0 as implemented in the compomics-utilities package (73). A phosphoRS score above was considered a confident localization. MSMS spectra of dimethylated arginine were manually inspected for neutral loss of dimethylamine (45.06 Da) and neutral loss of methylamine (31.04 Da) to distinguish between asymmetric dimethylarginine (aDMA) and symmetric dimethylarginine (sDMA), respectively.

## RESULTS

### Production of reporter NAF1, NHP2, GAR1 and SHQ1 cell lines

To better characterize H/ACA RNP biogenesis, we constructed stable and inducible HEK293 Flp-In T-REx cell lines by site-specific Flp-In integration. The expression of NAF1, NHP2, GAR1 or SHQ1 fused to Green Fluorescent Protein (GFP) or the Flag tag was induced by the addition of Doxycyclin (Dox) antibiotic in the culture medium. The fusion proteins were expressed at reduced or similar levels to that of endogenous ones if their expression was induced for a few hours, and generally at a higher level if their expression was induced overnight (Supplementary Figure S1). We usually observed lower cellular levels of Flag-tagged proteins compared to GFP-tagged proteins for the same time of induction of their expression. The GFP-tagged proteins may be more stable in cells than the Flag-tagged ones. We also observed, quite unexpectedly, that the expression of tagged-NHP2 or tagged-GAR1 in cells led to reduced levels of the corresponding endogenous proteins (see Supplementary Figure S1C and F for relevant examples). This observation was not made for SHQ1 and NAF1. To us, this suggested that cells somehow regulate the overall amount of the H/ACA core proteins in a yet-to-determine mechanism. This regulation may be important to insure the 1:1 stoichiometry of the four core proteins that is required for the integrity of the H/ACA RNPs.

### Quantitative proteomics revealed new unexpected associations between H/ACA core proteins, NAF1 and SHQ1

**Figure 1.**
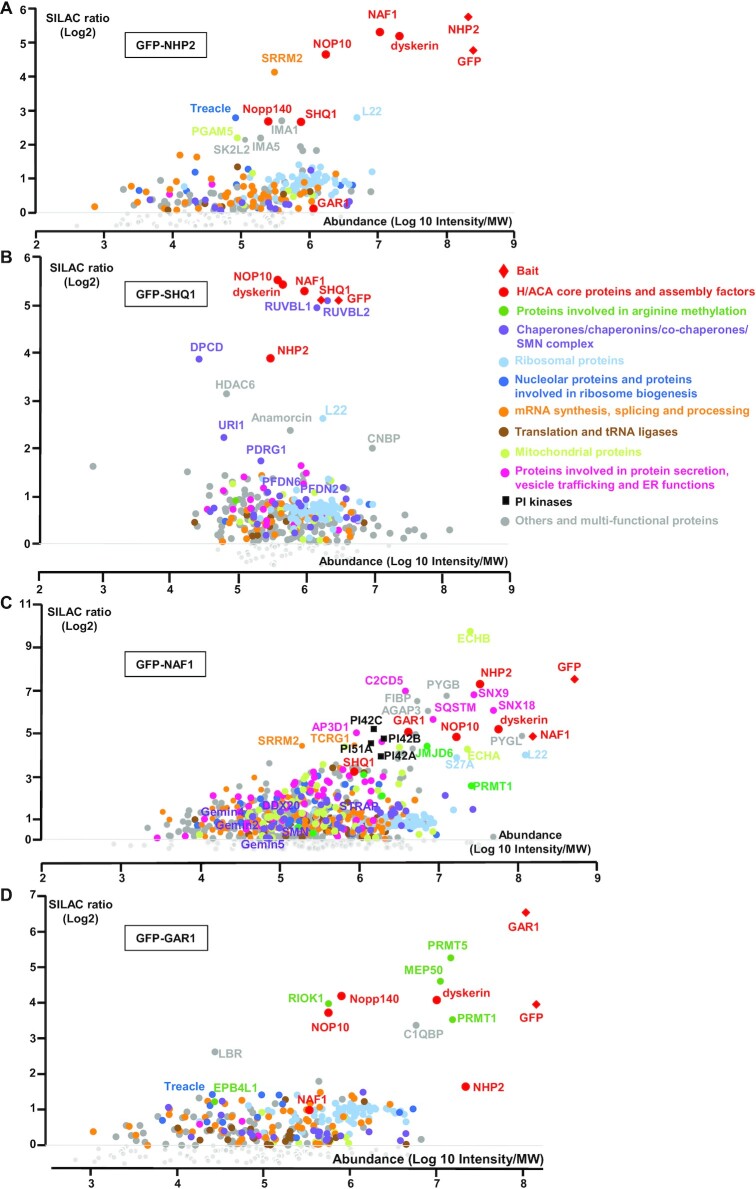
Analysis of the NHP2, SHQ1, NAF1 and GAR1 associated proteins by quantitative proteomic. Proteomic analysis of the partners of GFP-NHP2 (**A**), GFP-SHQ1 (**B**), GFP-NAF1 (**C**) and GFP-GAR1 (**D**). The graphs display SILAC ratios (y-axis, specific versus control IP done with parental HEK293 Flp-In T-REx cells) as a function of signal abundance (x-axis, log_10_(intensity)/MW). Each dot represents a protein. The labeled dots were arbitrarily selected to highlight proteins relevant to this study, and families of proteins associated with GFP-tagged proteins. The analysis of the functions of the associated proteins was performed using the Gene Ontology Resource and Uniprot. The full hit list with Significance B values is given in Supplementary Table S1.

We then performed stable isotope labeling by amino acids in cell culture (SILAC) proteomic experiments using GFP-NHP2, GFP-NAF1, GFP-SHQ1 or GFP-GAR1 proteins as bait. GFP tag has been chosen since it was shown to exhibit minimal non-specific binding to mammalian cell proteins, as compared to other tags, and it is also very efficient for quantitative IP-SILAC experiments (74). The expression of GFP-tagged proteins was induced for 3h. For SILAC analysis, following differential labeling of GFP-tagged protein-expressing cells and control cells with differently labeled isotopic amino-acids, whole-cell extracts were immunoprecipitated (IP) with anti-GFP antibodies, and pellets were subjected to quantitative mass-spectrometry analysis (Figure 1 and Supplementary Table S1). All the detected associations did not occur probably within the same complexes. The low abundance of some of the associations compared to others certainly reflected their transient and/or partial nature during the H/ACA RNP assembly or function.

Our IP-SILAC analysis showed that among the proteins associated with high abundance and specificity with GFP-NHP2, the top hits were NAF1, dyskerin, and NOP10 (Figure 1A). Interestingly, GAR1 was not significantly present in the GFP-NHP2 proteome in contrast to the two other core proteins, NOP10 and dyskerin, suggesting that GFP-NHP2 did not assemble into mature H/ACA RNP particles in these experimental conditions. In contrast and surprisingly, SHQ1 was efficiently associated with GFP-NHP2, even though with lower abundance than NAF1, dyskerin and NOP10. The analysis of the GFP-SHQ1 proteome indicated that GFP-SHQ1 was well associated with dyskerin as expected (22,35,36), and was also indeed surprisingly able to associate with NHP2, NOP10 and NAF1, all with high SILAC ratios and high abundance (Figure 1B).

Moreover, we observed that GFP-NAF1 was associated with the 4 H/ACA core proteins and SHQ1 (Figure 1C). The newly observed associations between NAF1 and GAR, and between NAF1 and SHQ1, were less abundant than the already reported associations of NAF1 with NHP2, NOP10, and dyskerin (Figure 1C). IP-SILAC analysis using GFP-GAR1 as the bait confirmed the formation in cells of an association between GAR1 and NAF1, even though this association was less abundant than the ones of GFP-GAR1 with the other H/ACA core proteins dyskerin, NOP10 and NHP2 (Figure 1D).

**Figure 2.**
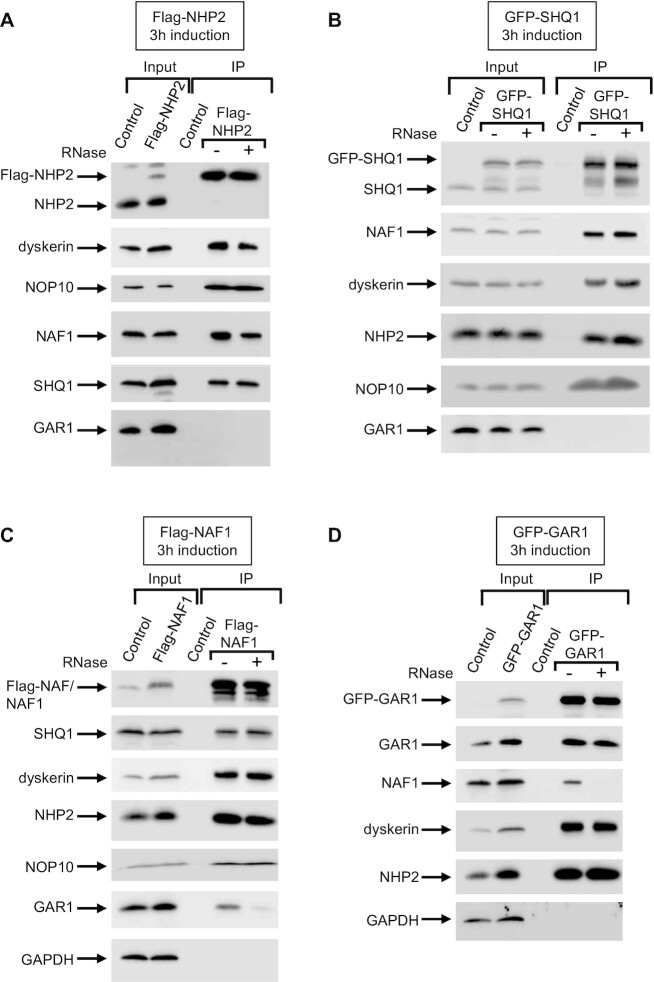
Co-IP assays. IPs were carried out on extracts from parental HEK293 Flp-In T-REx cells (Control) and HEK293 Flp-In T-REx cells expressing Flag-NHP2 (**A**), GFP-SHQ1 (**B**), Flag-NAF1 (**C**) and GFP-GAR1 (**D**) for 3 h, in the presence (+) or absence (–) of RNase A. The immunoprecipitated proteins were analyzed by SDS-PAGE and WB with antibodies to the indicated proteins; 5% of the inputs are shown (Input).

These associations observed in the IP-SILAC analyses between the H/ACA core proteins, and the two assembly factors SHQ1 and NAF1, were confirmed by inducing the expression of Flag- or GFP-tagged proteins for 3h and by analyzing their associated proteins by IP (Figures 2 and Supplementary Figure S2). The immunoprecipitated proteins (IP) and proteins from an aliquot of the respective extracts (Input) were fractionated by SDS-PAGE and analyzed by western blot (WB). An example of each IP is shown (Figure 2), and the quantifications of 4–6 different experiments are presented in graphs (Supplementary Figure S2). To determine the contribution of RNAs in these associations, we repeated the co-precipitation analysis in the presence of RNAse A. The absence of RNAs did not lead to statistically valid modifications of the observed associations, except for the one between NAF1 and GAR1 which was strongly reduced (Figures 2C, D, and Supplementary Figure S2C, D). In addition, we observed that untagged endogenous GAR1 was immunoprecipitated with GFP-GAR1 in an RNA-independent manner (Figure 2D and Supplementary Figure S2D). To us, this observation agrees (i) with the incorporation of at least a fraction of GFP-GAR1 proteins in mature bipartite H/ACA RNPs, containing one example of GFP-GAR1 and one example of untagged GAR1 and (ii) with the proposed direct interaction between the two heterotetramers of core proteins within the mature H/ACA RNP (17,18). Hence, all the interactions we have detected in our experiments could occur through the duplex of the core heterotetramer.

To confirm some of the results with endogenous proteins, we immunoprecipitated HEK293 cell extracts with an anti-NHP2 antibody that is efficient for this type of experiment (Supplementary Figure S2E). The immunoprecipitated proteins (IP) and proteins from an aliquot of the respective extracts (Input) were fractionated by SDS-PAGE and analyzed by WB. The anti-NHP2 antibody efficiently immunoprecipitated the three other H/ACA core proteins, indicating that the entire H/ACA RNP particle can be co-immunoprecipitated with NHP2 in these experimental conditions. Importantly, SHQ1 was also co-immunoprecipitated showing that endogenous NHP2 associates with SHQ1 as well. The rate of association was very low but reproducible, certainly reflecting the transient nature of the NHP2/SHQ1 association during H/ACA assembly.

### Stalled H/ACA assembly intermediates that do not contain GAR1

**Figure 3.**
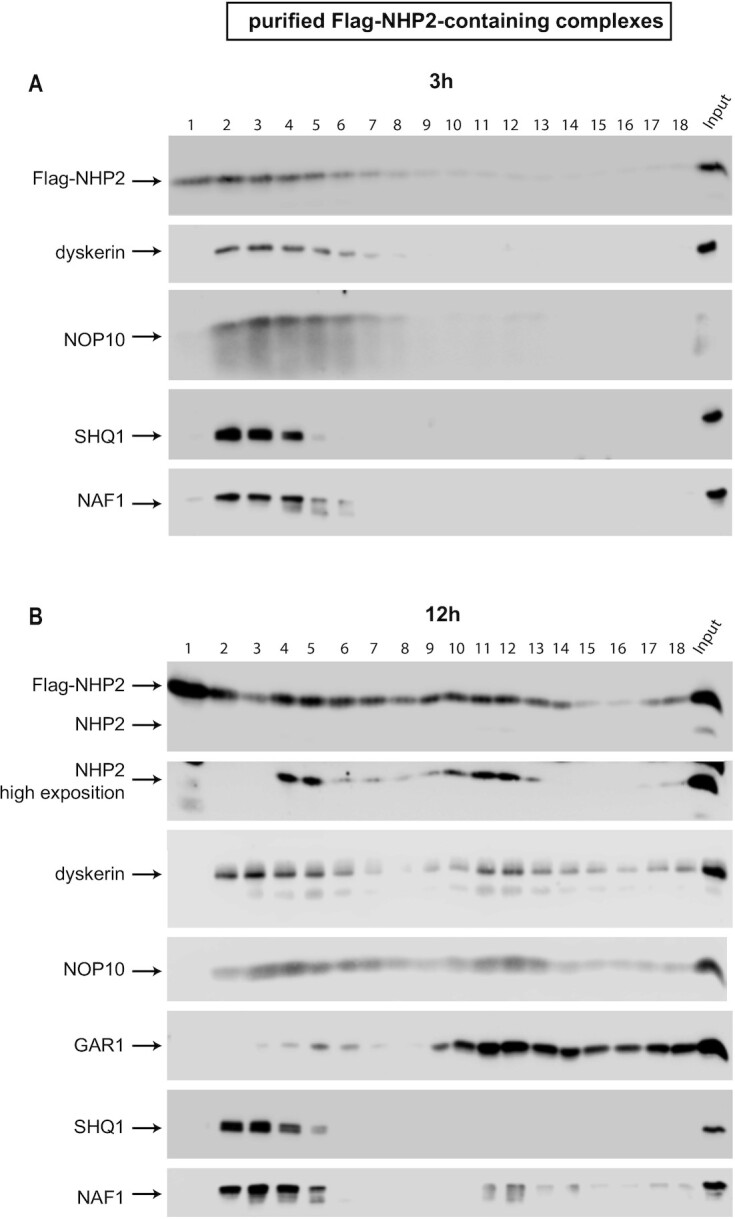
Sedimentation on glycerol gradients of purified Flag-NHP2-containing complexes. IPs were carried out on extracts from HEK293 Flp-In T-REx cells expressing Flag-NHP2 for 3 h (**A**) or 12 h (**B**). The immunoprecipitated proteins were eluted from the beads with an excess of flag peptides and fractionated on 10–30% glycerol gradients. The gradient was divided into 18 fractions. The proteins of each fraction were fractionated on SDS-PAGE and analyzed by WB with antibodies to the indicated proteins. Lane numbers correspond to fraction numbers. Fraction 1, the top of the gradient; fraction 18, the bottom of the gradient; Input, 10% of the unfractionated purified complexes.

In addition to the absence of GAR1 in the tagged-NHP2 immunoprecipitate (Figures 1A and 2A), we also did not observe the association of endogenous NHP2 with tagged-NHP2 in our experimental conditions (Figure 2A). The expression of tagged NHP2 for only 3h was not sufficient to incorporate some of this protein into mature bipartite particles (while it was done after 12 h of expression, see in the next chapter). The steric hindrance due to the tag present on NHP2 may delay the formation of the mature bipartite H/ACA RNPs. This offered the opportunity to approach experimentally assembly steps otherwise inaccessible using ‘stalled’ particles. Hence, we purified and analyzed these Flag-NHP2-containing complexes. To do so, Flag-NHP2 expressed for 3h was immunoprecipitated using anti-Flag antibodies on agarose beads. The immunoprecipitated complexes were eluted from the beads with an excess of Flag-peptides, concentrated, and fractionated on a 10 to 30% glycerol gradient (Figure 3A). Proteins of each fraction were separated by SDS-PAGE and their sedimentation patterns were analyzed by WB. It is important to note that different complexes of different compositions can have the same coefficient of sedimentation and therefore be present in the same fraction of the gradient.

In parallel, total cell extract from the HEK293 Flp-In T-REx cell line was also fractionated on a 10–30% glycerol gradient, to analyze the pattern of sedimentation of the endogenous H/ACA proteins in a total extract, and the ones of several proteins used as markers (Supplementary Figure S3). The *S* value markers at the bottom of the Figure are derived from the migration behavior of the characterized U1 snRNP (12S), and of the ribosomal 40S and 60S particles, revealed by the analysis of U1A, L5, and S6 proteins, respectively. The peaks of sedimentation of GAR1 and fibrillarin were observed for fractions 4 to 6 and corresponded to the known 10S-15S sedimentation values for mammalian H/ACA and C/D snoRNPs (75–78). Interestingly, the sedimentation behavior of NHP2, dyskerin and NOP10 was slightly different from GAR1. Indeed, in addition to being present in fractions 4–6, these proteins were also sedimented in high amounts in lower fractions 2 and 3, suggesting that they can be incorporated into cellular stable lower-molecular weight complexes that seem to not contain GAR1 (see also below). The peaks of sedimentation of NAF1 and SHQ1 was located in fractions 3–4, but these proteins were also present in fractions 2 and upper fractions. All proteins extended farther into the gradient, likely due to their association in higher-order complexes.

The sedimentation profile of purified Flag-NHP2-containing complexes after 3h of expression is shown in Figure 3A. A fraction of the tagged protein sedimented in the top 1 fraction of the gradient and therefore was likely free in the cells. Most of the Flag-NHP2-containing complexes were present in fractions 2 to 7, together with NOP10 and dyskerin. NAF1 and SHQ1 were also mostly present in the complexes that sedimented in fractions 2 to 5. Therefore, our data indicated unexpectedly that the 5 proteins: Flag-NHP2, dyskerin, NOP10, SHQ1 and NAF1 were likely all present in the same complexes. Several distinct complexes containing these 5 proteins may exist since they sedimented in 4 different fractions, the complexes with a higher coefficient of sedimentation likely also containing other still unknown proteins.

### Formation of multiple flag-NHP2-containing complexes

To test the possibility that the tag present on NHP2 delayed the formation of the H/ACA RNPs, the expression of Flag-NHP2 was induced for a longer time (12h) before doing the IP (Supplementary Figure S4A, B). In these conditions, Flag-NHP2 was well associated with GAR1 in all experiments done. We observed that (i) the level of Flag-NHP2 (after 12 h of induction) over the level of untagged NHP2 was not the same in each experiment (compare the Input in the two examples, Supplementary Figure S4A and B) and (ii) the level of untagged NHP2 co-immunoprecipitated with Flag-NHP2 depended on the cellular level of Flag-NHP2. Indeed, untagged NHP2 was better co-immunoprecipitated when the level of Flag-NHP2 was lower than the untagged one than when the level of Flag-NHP2 was higher than the untagged one (compare the IP, Supplementary Figure S4A and B). Hence, after 12 h of expression, at least some Flag-NHP2 seemed to be incorporated into H/ACA RNPs containing GAR1, and either two Flag-NHP2 or one Flag-NHP2 and one NHP2. We observed that the association of Flag-NHP2 with untagged NHP2 was at least partially dependent on the presence of RNA (Supplementary Figure S4A–C). Next, we studied the cellular localization of GFP-NHP2 by analyzing the direct fluorescence of the GFP, after 3 or 12 h induction of expression (Supplementary Figure S4D). After 12 h induction, GFP-NHP2 was located in the nucleoplasm, but also in the nucleolus and the CBs, as shown by the co-localization experiment with nucleolin and coilin, respectively. This observation agreed with the incorporation of some of the expressed tagged NHP2 into mature H/ACA RNPs. In contrast, after 3 h of induction, GFP-NHP2 displayed a different localization. Even though the level of expression was low for this induction time, we could observe that GFP-NHP2 accumulated more throughout the nucleoplasm and did not accumulate into CBs. This agreed with the incorporation of GFP-NHP2 in stalled intermediate complexes (see above the analysis of purified complexes). However, even though GFP-NHP2 did not associate with GAR1 and therefore did not form mature H/ACA RNPs in these experimental conditions, a fraction of GFP-NHP2 was surprisingly nevertheless addressed to the nucleolus (Supplementary Figure S4D). This nucleolar fraction of the GFP-NHP2/NOP10/dyskerin complexes might still be associated with NAF1. We cannot also exclude the possibility that a very low level of GAR1 undetectable by IP/WB was associated with a fraction of GFP-NHP2.

We analyzed the purified Flag-NHP2-containing complexes after 12h of induction of the tagged protein expression on a 10–30% glycerol gradient as described above for 3h of expression (Figure 3B). The level of untagged NHP2 being very low in the immunoprecipitate, an over-exposition of the WB for this protein is also shown (NHP2 high exposition). We observed that the Flag-NHP2-containing complexes were more dispersed throughout the gradient than after 3h of expression (compare Panels A and B, Figure 3), indicating the presence of multiple different complexes in the immunoprecipitated with different compositions. Indeed, several distinct complexes can be observed: (i) Flag-NHP2 complexes also containing SHQ1 and NAF1 were present in fractions 2–5. Some of these complexes were likely the same as the ones observed after 3h of induction of the expression of Flag-NHP2, i.e. Flag-NHP2/dyskerin/NOP10/SHQ1/NAF1 complexes (Figure 3A); (ii) Flag-NHP2 complexes containing also GAR1 and untagged NHP2 were detected in fractions 4 and 6. Some of them should correspond to mature H/ACA RNPs (75–78) (see also Supplementary Figure S3); (iii) Flag-NHP2 containing complexes of higher sedimentation values were also formed, some certainly containing also the 3 other core proteins and NAF1 since all these proteins sedimented in the same fractions, but not SHQ1. These complexes may be higher-order intermediate assembly complexes. Some of them seemed to contain also untagged NHP2 (complexes sedimented in fractions 7 to 13, and fraction 18) indicating that these complexes contained two core-proteins heterotetramers. One of these complexes was particularly accumulated in fractions 11 and 12. Untagged NHP2 was not detectable in fractions 14 to 17, but the low level of this protein present in the immunoprecipitate may not allow its detection by WB in these fractions. Thus, we observed that multiple complexes containing the core proteins (present as a heterotetramer or as a dimer of heterotetramer), NAF1, SHQ1, and other proteins, are present in cells, and might be formed successively during H/ACA RNP assembly.

It is interesting to note that only a small fraction of Flag-NHP2 seemed to be incorporated into mature H/ACA RNPs. Therefore, even after 12h of expression of the tagged-NHP2, intermediate complexes of the H/ACA RNP assembly seemed to accumulate, strongly suggesting that the tag present on NHP2 somehow delayed or partially prevented the formation of the mature bipartite H/ACA RNPs.

### Analysis of flag-NAF1-containing complexes

**Figure 4.**
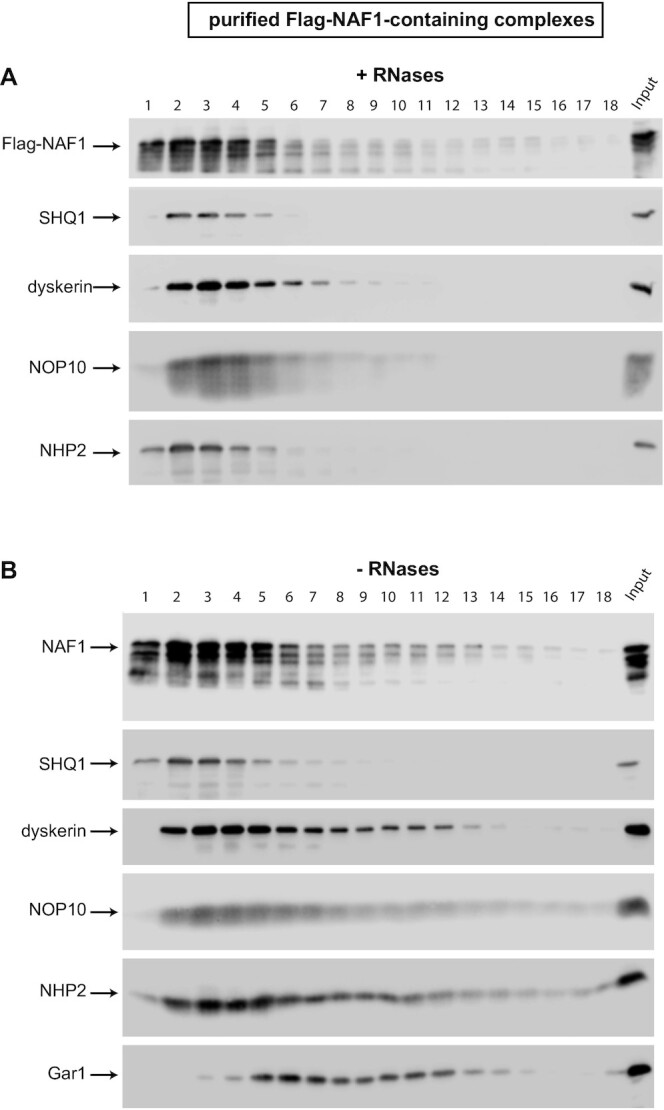
Sedimentation on glycerol gradients of purified Flag-NAF1-containing complexes. IPs were carried out on extracts from HEK293 Flp-In T-REx cells expressing Flag-NAF1 in the presence (**A**) or absence (**B**) of RNases. The legend is as in Figure 3.

To extend our studies, we also purified Flag-NAF1-containing complexes in the presence (Figure 4A) or the absence (Figure 4B) of RNases and analyzed them on glycerol gradients. In both conditions, Flag-NAF1 was present in most of the fractions reflecting its association with numerous proteins (Figure 1C) and therefore its capacity to form multiple complexes in cells. As for Flag-NHP2, some Flag-NAF1 was also present in fraction 1, which certainly represented free Flag-NAF1.

The complexes purified in the presence of RNases mostly sedimented in fractions 2–5, and also contained SHQ1, dyskerin, NOP10 and NHP2 (Figure 4A). We hypothesized that these complexes were the same ones purified using Flag-NHP2 expressed during 3h as bait (Figure 3A). This observation reinforced the hypothesis of the RNA-independent formation of dyskerin/NHP2/NOP10/SHQ1/NAF1 complexes. Moreover, in the absence of RNases, additional Flag-NAF1-containing complexes with upper coefficients of sedimentation were purified, containing certainly also GAR1 and the other core proteins (Figure 4B).

### Proteomic analysis revealed new proteins associated with NHP2, GAR1, SHQ1 and NAF1

Our IP-SILAC experiments revealed that, apart from the proteins already involved in H/ACA RNP biogenesis and from the C/D and H/ACA RNP chaperone Nopp140, numerous proteins were additionally associated with GFP-tagged proteins, some of them with high efficiency and specificity.

Indeed, we observed a strong association of GFP-SHQ1 with RUVBL1 and RUVBL2 (Figure 1B, Supplementary Table S1). Four of the components of the Prefoldin-like module (PFDL): URI1, PDRG1, PFDN6 and PFDN2, were also associated with SHQ1. The chaperone complex R2TP associates with the PFDL to form the PAQosome involved in the quaternary structure assembly of many cellular complexes (6,79). Our observation raises the possibility that the entire PAQosome can play a role in the assembly/disassembly of some of the H/ACA intermediate complexes during H/ACA biogenesis. DPCD, which was also well associated with GFP-SHQ1, might also intervene since this protein has been proposed to be a co-factor of the R2TP (65,80,81).

Both PRMT1 and PRMT5, the predominant type I and II protein arginine methyltransferases, were among the proteins strongly associated with GFP-GAR1 in our proteomic analysis (Figure 1D; Supplementary Table S1). Protein arginine methyltransferases (PRMTs) catalyze the widespread protein arginine methylations (for review, 82). RIOK1 and MEP50, two PRMT5 co-factors (82–87), were also associated with GFP-GAR1 in our IP-SILAC analysis (Figure 1D; Supplementary Table S1), suggesting that the PRMT5/RIOK/MEP50 complex specifically methylates GAR1.

Finally, numerous proteins were associated with GFP-NAF1, some of them with high efficiency and specificity (Figure 1C; Supplementary Table S1). This is the case for four phosphatidylinositol kinases, a large number of mitochondrial proteins, and proteins involved in vesicle trafficking and protein secretion. These observations indicate that NAF1 may have multiple functions in cells besides the biogenesis of H/ACA RNPs.

### GAR1 contains both mono-methylated and dimethyl arginines, that favor GAR1 association with NHP2

**Figure 5.**
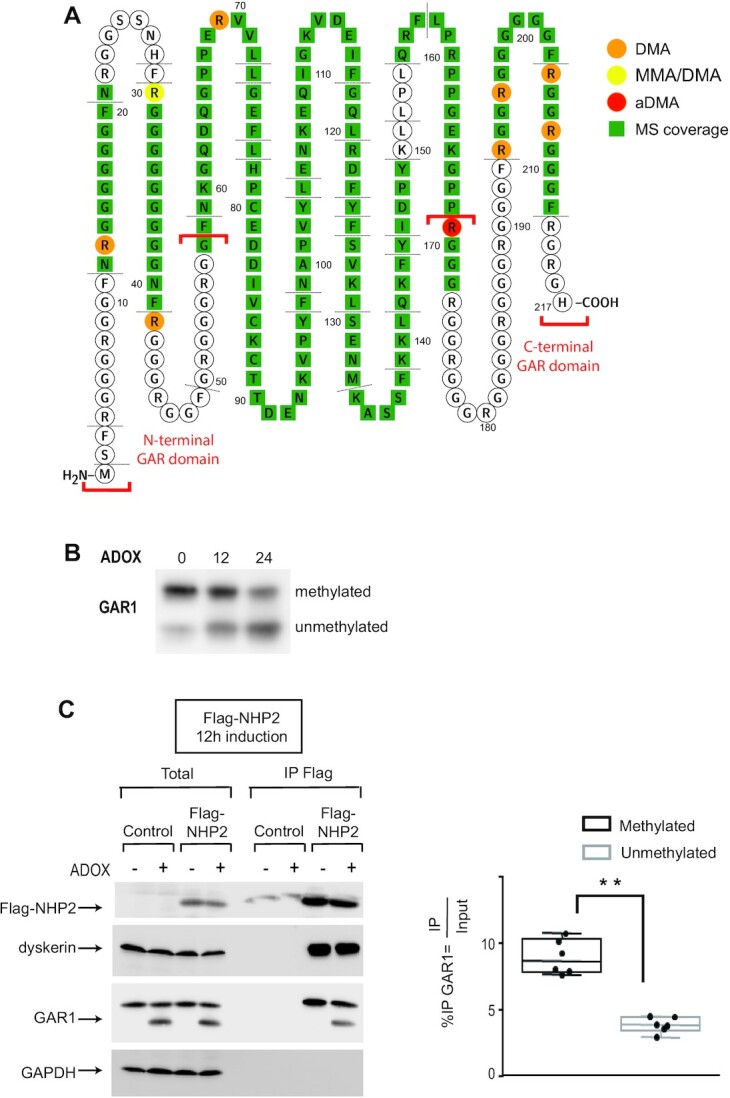
Identification of arginine methylations in GAR1 by MS/MS and influence of these modifications on GAR1 association with the other H/ACA core proteins. (**A**) The region covered in our MS/MS analysis is highlighted in green within the sequence of human GAR1. The methylated arginines that we identified in our study are circled in red, yellow, or orange depending on the nature of the methylation. DMA: di-methylated arginine; aDMA: asymmetrical di-methylated arginine; MMA mono-methylated arginine. (**B**) Inhibition of methylation in cells leads to an increased level of the unmethylated form of GAR1. HEK293 cells were not treated (0) or treated for 12 or 24 h with ADOX. Extracts were prepared and analyzed on SDS-PAGE and WB with GAR1 antibody. The upper band corresponds to methylated GAR1 and the lower band to unmethylated GAR1. (**C**) ADOX treatment modifies GAR1 association to NHP2. Parental HEK293 Flp-In T-REx cells expressing Flag-NHP2 for 12 h were not treated (–) or treated (+) with ADOX for 24 h. IPs were carried out on extracts from these cells using anti-Flag antibodies. The immunoprecipitated proteins were analyzed by SDS-PAGE and WB with antibodies to the indicated proteins; 5% of the inputs are shown (Input). WB signals were quantified using Fusion Solo (Vilber). Boxplots (black boxes, methylated GAR1; grey boxes, unmethylated GAR1) represent the association of both forms of GAR1 with Flag-NHP2 over the Input obtained after ADOX treatment. Kolmogorov-Smirnov tests were calculated from 6 independent experiments; significant changes are indicated: ** (*P*< 0.01).

GAR1 is a small protein consisting of two glycine-arginine-rich (GAR) domains, also called RGG/RG motifs, that flank either side of the central core (88) (Figure 5A). RGG/RG motifs are preferred sites for arginine methylation in proteins (for review, 82). The GAR domains of GAR1 have been thought to contain arginine methylations for a long time as the yeast Gar1p protein can be methylated *in vitro* and *in vivo* by Hmt1p, the homolog of PRMT1 (89,90). However, the nature of the methylations and their localization within the yeast or mammalian GAR1 proteins are not well known. Recently, cell proteome-wide studies of endogenous arginine methylation sites by mass spectrometry analysis have been developed (91–96). We analyzed the raw data available from the literature, obtained after the concentration of the methylated peptides by high pH strong cation exchange (SCX) or by immunoaffinity purification using monomethyl or dimethyl arginines antibodies. This analysis allowed us to list several methylated arginines in human GAR1 (Table 1) (91–96). To complete these data, we examined the methylation status of purified human GAR1. To do so, GFP-GAR1 was expressed in HEK293 Flp-In T-REx GFP-GAR1 expressing cells, immunoprecipitated with anti-GFP antibody from cell total extract in high salt conditions to disrupt its interactions with other proteins. After IP, GFP-GAR1 was fractionated on SDS-PAGE, eluted from the gel, digested with chymotrypsine, and analyzed by MS. As modifications of lysine and arginine-like methylations often block digestion by trypsin, alternative enzymes for digestion were tested *in silico* using peptide mass software (https://web.expasy.org). Theoretical chymotrypic peptides with no missed cleavage and masses between 750 and 4000 Da ideally cover 90% of the GAR1 sequence in the theoretical digest. Experimental peptides identified in the LC-MS/MS experiment cover 60% of the proteins (in green in Figure 5A). DMAs were identified at 9 different positions, 8 of them being located in the GAR domains. Manual inspection of MS/MS spectra revealed neutral loss specific for aDMA (dimethylamine, 45.06 Da) for R169-containing peptide. The absence of neutral loss specific for sDMA or aDMA in the MS/MS spectra of the other dimethylated arginine-containing peptides did not allow the distinction between these two types of dimethylations for the other positions. The analysis of the spectra revealed the presence of both mono-methylated and dimethylated forms of R30 in purified GAR1.

**Table 1.** Positions of the methylated arginines in GAR1. DMA: di-methylated arginine; aDMA: asymmetrical di-methylated arginine; MMA mono-methylated arginine. References are indicated, as well as methylated arginines identified in this study that are marked by a star

Position	Nature of the methylation	References
8	MMA	(96)
13	MMA	(96)
	DMA	This study
22	MMA	(95,96)
30	MMA	(94,96)
	MMA/DMA	This study
42	MMA	(96)
	DMA	This study
69*	DMA	This study
169*	aDMA	This study
185	MMA	(95)
194*	DMA	This study
197	MMA	(94)
	aDMA	(91)
	DMA	(92), this study
205	MMA	(96)
	DMA	(92), this study
208	DMA	(92,93), this study
213	DMA	(93)

It was previously shown that *in vitro*-translated GAR1 migrated as a doublet in SDS–PAGE system, the faster and the slower migrating GAR1 bands being respectively the unmethylated and methylated forms of GAR1 (23). We treated HEK293 cells for 12 or 24 h with ADOX, an inhibitor of methylases, fractionated the extract on SDS-PAGE, and analyzed GAR1 by WB (Figure 5B). We also observed a faint faster migrating band in untreated cells. The intensity of this band increased with the time of ADOX treatment, confirming that the faster migrating band corresponds to unmethylated GAR1. To test whether the unmethylated form of GAR1 is integrated into the H/ACA RNP complexes, we analyzed the association of methylated and unmethylated forms of GAR1 with Flag-NHP2 protein in HEK293 Flp-In T-REx cell lines after a 24 h treatment with ADOX and a 12h induction of the expression of the tagged protein (Figure 5C). We observed that the unmethylated form of GAR1 was less associated with Flag-NHP2 than methylated ones, which could indicate a moderate and/or indirect effect of methylation on GAR1 fate.

## DISCUSSION

### Formation of early protein-only assembly intermediates

**Figure 6.**
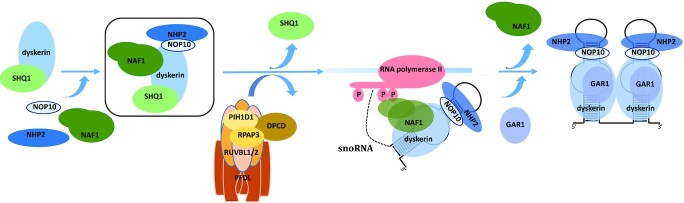
Schematic model for box H/ACA RNP assembly. The early assembly intermediate proposed in this study is framed in black.

Overall, our IP-SILAC and IP-WB experiments using NAF1, NHP2, and SHQ1 as baits, as well as the analysis on glycerol gradient of purified Flag-NHP2 and Flag-NAF1 containing complexes, indicate that numerous complexes containing the H/ACA core proteins, SHQ1 and NAF1 exist in cells. Our observations indicate that several intermediate complexes are formed successively during H/ACA RNP assembly. More experiments will be required to characterize all of them. Previous works showed clearly that NOP10 interacts directly with both NHP2 and dyskerin forming a core trimer, and NAF1 joins this trimer by directly interacting with the GAR1-interacting domain of dyskerin (17–19,21,22,40,41). Moreover, it was shown that SHQ1 interacts directly with the RNA binding domain of dyskerin (22,35,36). Our data revealed unexpectedly that these five proteins (dyskerin, SHQ1, NAF1, NOP10 and NHP2) can be present within the same complexes independently of the presence of RNAs. Therefore, we propose that protein-only pre-complexes containing at least these five proteins are formed during H/ACA RNP assembly in mammalian cells (the pentamer complex in the black square, Figure 6). We cannot exclude the possibility that the proposed complexes can be formed after cell lysis. However, in agreement with the evolutionarily conserved formation of such complexes in eukaryotic cells, early studies with yeast proteins revealed the association of Shq1p with Naf1p and Nhp2p in a Y2H screen and by *in vitro* pull-down assays in reticulocyte lysates (31,97). Moreover, a Cbf5p/Shq1p/Naf1p/Nop10p/Nhp2p complex can be formed *in vitro* using purified yeast proteins (98). Purified complexes containing these five proteins sedimented in several fractions on glycerol gradients (Figures 3A and 4A), indicating that they can form several different complexes with still unknown proteins.

Using elegant *in vitro* translation-immunoprecipitation assay in reticulocyte lysates and *in cellulo* co-tethering experiment, Grozdanov *et al.* (35) previously observed that SHQ1 interacts with dyskerin alone, but not with dyskerin in the context of the core trimer dyskerin/NOP10/NHP2 or when dyskerin is in a complex with NAF1. Then the interaction of SHQ1 with dyskerin may be a prerequisite to its subsequent association with NOP10, NHP2 and NAF1 (Figure 6). SHQ1 is involved in the initial folding of dyskerin and in preventing dyskerin illicit binding to RNAs, by interacting with the RNA binding domain of dyskerin (35,36,98). For these reasons, it has been proposed that SHQ1 interaction with dyskerin occurs in the cytoplasm concomitantly to or just after dyskerin translation (35). The binding of NHP2, NOP10, and NAF1 to the pre-formed SHQ1/dyskerin complex could occur in either the cytoplasm or the nucleus.

CryoEM analysis of purified mammalian telomerase enzyme recently proposed that the two core-proteins heterotetramers present on mature bipartite H/ACA RNPs directly interact through the formation of a dyskerin/dyskerin dimer (17,18). Our IP-WB analyses of mature H/ACA RNPs using GFP-GAR1 as bait indeed revealed that GFP-GAR1 was associated with untagged GAR1 in an RNA-independent manner (Figure 2). This observation indeed agrees with the formation of a dimer of heterotetramers in the mature H/ACA RNPs. In contrast, the association of Flag-NHP2 (after 12 h of its expression) with untagged NHP2 was partially dependent on the presence of RNA (Supplementary Figure S4A). However, to our opinion, it is difficult to conclude that this observation disagrees with the formation of a dimer of heterotetramers in the mature H/ACA RNPs, since Flag-NHP2 was only slightly incorporated into mature H/ACA RNPs in these experimental conditions (Figure 3B).

If a dyskerin-dyskerin interaction is formed, this interaction may occur before the association with the H/ACA RNA or concomitantly with the binding of the proteins to the H/ACA RNA. We observed that endogenous SHQ1 did not associate with GFP-SHQ1 (Figure 2), and NAF1 did not associate with GFP-NAF1 (Data not shown). It is therefore improbable that a dimer of the proposed pentamer exists. However, we cannot exclude that a dimer of NOP10/dyskerin/NHP2 is formed through a direct dyskerin/dyskerin interaction, with one dyskerin bound to SHQ1 and the other one to NAF1.

### NAF1 and GAR1 are both present in the same complexes

Our experiments also indicated that GAR1 and NAF1 are present within the same complexes during H/ACA RNP assembly. This was surprising since these two proteins share the same binding site on dyskerin, and therefore the release of NAF1 is a prerequisite for the binding of GAR1 to dyskerin (33,41). In agreement with our observation, an association between Naf1p and Gar1p has also been observed previously by IP experiments in yeast cells (32,39). One possibility is that, during the H/ACA RNP assembly, the exchange between NAF1 and GAR1 does not occur concomitantly on both H/ACA hairpins. Therefore, one molecule of dyskerin can still be bound to NAF1 while the other is already bound to GAR1. However, we showed that the GAR1/NAF1 association is mediated by RNA. If dyskerin forms a homodimer, pulling down one molecule of dyskerin may bring down the other one with its associated proteins even in the presence of RNases. It is therefore possible that GAR1 interacts transiently with the H/ACA RNA bound to the dyskerin/NOP10/NHP2/NAF1 complexes, either directly or indirectly through a still uncharacterized bridging factor.

The mechanism catalyzing the conformational switch between NAF1 and GAR1 is still debated (22,41). The SMN complex has been proposed to play a role in this remodeling event (58). Some components of the SMN complex were only detected slightly above the background in the GFP-NAF1 IP-SILAC experiment (Figure 1C). In cells transfected by siRNAs specific for SMN or Gemin3, we did not observe a defect in GAR1 association to H/ACA pre-RNPs, nor a defect in NAF1 release (Data not shown). The level of remaining SMN complexes may be sufficient to allow the association of GAR1 to the pre-RNPs and the exchange with NAF1. Another possibility is that the SMN complex is not required for this step of the assembly. In the future, it will be interesting to test whether some of the proteins newly identified in the GAR1 SILAC-IP experiment can play a role in the GAR1/NAF1 exchange.

### NAF1, GAR1, and NHP2 proteome analysis reveal new putative factors involved in H/ACA RNP assembly or function

Here, we report an extensive proteomic analysis of the proteins associated in mammalian cells with two core components of H/ACA RNPs, GAR1 and NHP2, and two H/ACA assembly factors, SHQ1 and NAF1. Some of the identified proteins were specifically associated with SHQ1, NAF1, NHP2, or GAR1, others were present in several proteomes (Supplementary Figure S5). Besides H/ACA core proteins, factors known to be involved in H/ACA biogenesis (NAF1, SHQ1), and the C/D and H/ACA RNP chaperone Nopp140, the majority of the proteins associated with GAR1 and NHP2 were proteins involved in RNA metabolism (ribosomal proteins, U snRNP components, proteins implicated in mRNA biogenesis, translation, ribosome production) (Figure 1, Supplementary Table S1). In contrast, NAF1 also associates with a plethora of proteins not involved in RNA metabolism, such as mitochondrial proteins, and proteins involved in vesicle trafficking, protein secretion, endoplasmic reticulum composition and functions, phosphorylation of phosphatidylinositol, etc., indicating that NAF1 has multiple and diverse functions in cells besides its role in H/ACA RNP assembly. Future investigations will be required to test whether the proteins present in the GAR1, NAF1, SHQ1 and NHP2 proteomes are required for H/ACA RNP biogenesis or functions, whether they are present in the same complexes or individual ones, and whether they interact directly or indirectly with the bait proteins.

Interestingly, IMA1 (importin α1) and IMA5 (importin α5) were strongly associated with tagged NHP2 (Figure 1A) and could be responsible for the nuclear import of the cytoplasmic pre-formed NHP2-containing complexes. The expression of tagged NHP2 for only 3 h led to the accumulation of tagged-NHP2 containing pre-complexes that are otherwise not detectable, allowing certainly the detection of IMA5 and IMA1 associated with GFP-NHP2 in our experiments.

It was previously shown that, in the nucleoplasm, SHQ1 is released from dyskerin by the action of the R2TP chaperone complex, constituted of the AAA + ATPases RUVBL1 and RUVBL2 associated with PIH1D1 and RPAP3 proteins (37,38). The release of SHQ1 occurs before or concomitantly with the association with nascent H/ACA RNAs (33,35,36,39,40,98,99). The R2TP forms the PAQosome with the Prefoldin-like module (PFDL) and is known to assemble the quaternary structure of many cellular protein–protein and RNA–protein complexes (6,79). Four of the six components of the PFDL were found to be strongly associated with SHQ1. Our observation suggests that the entire PAQosome can play a role in the assembly/disassembly of some of the H/ACA assembly intermediates during H/ACA biogenesis (Figure 6). DPCD is a strong binder of RUVBL1 and 2, and it has been proposed to be a co-factor of the AAA + ATPases even if its role remains elusive (65,80,81). Its strong association with SHQ1 raises the possibility that it modulates the R2TP/PFDL activity to help the release of SHQ1.

### Methylated arginines exist both in mono-methylated and di-methylated forms in GAR1 and they facilitate GAR1 association with the other core proteins

The analyses, available in the literature, of the cell proteome-wide studies of endogenous arginine methylation sites by MS revealed the presence of ten methylated arginines in GAR1 (Table 1) (91–96). Our analysis of arginine methylations within purified GAR1 confirmed the presence of methylated arginines at eight sites and revealed three new methylated arginines at positions 69, 169 and 197 (Figure 5 and Table 1). All these arginines were found to be dimethylated in our analysis, except at position 30 where mono and dimethylated forms coexist. In most of the previous whole-cell proteome analyses, methylated peptides were purified by immunoaffinity using monomethyl or dimethyl arginine antibodies before MS analysis to enrich specifically one form of arginine methylation. The comparison of the data obtained in these studies with our data indicates that most methylated arginines in GAR1 exist in cells in both monomethyl and dimethylated forms. This suggests that the status of the GAR1 methylations could be a way to regulate the function of this protein in the cells. Except at position 169 where our analysis of the MS/MS spectra demonstrated that the arginine is asymmetrically dimethylated, we were not able to distinguish whether the dimethylations were symmetrical and asymmetrical at the other positions.

Protein arginine methyltransferases are classified as types I, II and III. All types can catalyze the formation of monomethylarginine (MMA) on guanidine nitrogen atom of arginine residues. Type I enzymes can additionally catalyze the formation of asymmetric dimethylarginine (aDMA), by adding a second methyl group to the same nitrogen atom. Type II enzymes can catalyze the formation of symmetric dimethylarginine (sDMA), with a second methyl group added to the other terminal nitrogen atom. Type III PRMT activity is restricted to the generation of MMA. PRMT5 forms a hetero-octameric complex with MEP50 (83,84). The PRMT5/MEP50 complex binds various co-factors allowing the specific recruitment of distinct substrates, such as pICln or RIOK1 co-factors that are required respectively for Sm protein and coilin methylation (82,85–87). The observed strong associations of both PRMT1 and the PRMT5/RIOK1/MEP50 complex with GAR1 reflect that these two enzymes modify GAR1, and agree that both forms of dimethylations exist within GAR1. RIOK1 is predominantly cytoplasmic (85) which suggests that at least some of the GAR1 methylations occur in this compartment. We observed that unmethylated GAR1 was associated with NHP2, but less efficiently than methylated ones (Figure 5). Therefore, arginine methylations are not strictly essential for GAR1 integration in the H/ACA particle, but either facilitate this event and/or increase the stability of GAR1 within the RNPs. Moreover, while the core domain of GAR1 is sufficient and essential for pseudourydilation and substrate turnover in yeast reconstituted particles, the presence of the GAR domains increased the substrate turnover rate in the same yeast reconstituted particle, likely through transitive interactions of these domains of GAR1 with substrate RNAs (22). Therefore, GAR1 methylation may both facilitate H/ACA assembly and function.

## DATA AVAILABILITY

The mass spectrometry proteomics data have been deposited to the ProteomeXchange Consortium via the PRIDE (100) partner repository with the dataset identifiers PXD026747, PXD026749, PXD035330 and PXD026752.

## Supplementary Material

gkad129_Supplemental_Files
