# Supplementary material for: Proteomic analyses reveal new features of the box H/ACA RNP biogenesis
Source: Nucleic Acids Res. 2023 Mar 3;51(7):3357–74. doi: 10.1093/nar/gkad129 (PMC10123114; doi:10.1093/nar/gkad129)
Supplement: gkad129_Supplemental_Files [file gkad129_supplemental_files.zip › supplemental material.pdf]

ONLINE SUPPLEMENTAL MATERIAL

Supplemental Figure 1. **Expression analysis of tagged proteins in constructed HEK293 Flp-In T-REx cells.** Analysis of the expression of GFP-NAF1 (A), GFP-NHP2 (B), GFP-GAR1 (C), GFP-SHQ1 (D), Flag-NAF1 (E), Flag-NHP2 (F), and Flag-GAR1 (G) in HEK293 Flp-In T-REx cells in which corresponding ORFs were stably integrated, after induction of their expression by the addition of Doxycycline in the medium for 2, 4 or 12h. The proteins were analyzed by SDS-PAGE and WB with antibodies to the indicated proteins. GAPDH was used as a loading control.

Supplemental Figure 2. Co-IP assays. A-D/ WB signals obtained in Figure 2 were quantified using Fusion Solo (Vilber), except for NOP10 which migrated at the very bottom of the gels. Boxplots (black boxes, -RNase; grey boxes, +RNase) represent the association of each analyzed protein with the tagged proteins. Wilcoxon tests were calculated for the two distributions (-RNase, + RNase) of each condition from 4 to 6 independent experiments; significant changes are indicated: * ($p<0.05$). **E/** IPs were carried out on HEK293 total cell extracts using anti-NHP2 antibody bound to A-sepharose, in the presence (+) or absence (-) of RNAse A. A-sepharose alone was used as control. The immunoprecipitated proteins were analyzed by SDS-PAGE and WB with antibodies to the indicated proteins; 5% of the inputs are shown (Input). Quantification was done as above.

Supplemental Figure 3. Sedimentation analysis of H/ACA RNP proteins by glycerol gradients. Total cell extracts from HEK293 Flp-In T-REx cells were fractionated on 10-30 % glycerol gradients. The gradient was divided into 18 fractions. The proteins of each fraction were fractionated on SDS-PAGE and analyzed by WB. The migration pattern of U1A (protein of the U1 snRNP), L5 (protein of the large ribosomal subunit), and S6 (protein of the small ribosomal protein) indicates respectively the positions of 12S, 40S, and 60S. Lane numbers correspond to fraction numbers. Fraction 1, the top of the gradient; fraction 18, the bottom of the gradient; Input, 5% of the unfractionated total extract. Fractions 4 to 6 certainly containing C/D and H/ACA mature RNPs are framed in blue for GAR1 and fibrillarin.

Supplemental Figure 4. Co-IP assays using Flag-NHP2 expressed in cells for 12h as bait, and cellular localization of GFP-NHP2. A-B/ IPs were carried out on extracts from parental HEK293 Flp-In T-REx cells (Control) and HEK293 Flp-In T-REx cells expressing Flag-NHP2 for 12h, in the presence (+) or absence (-) of RNase A. The immunoprecipitated proteins were analyzed by SDS-PAGE and WB with antibodies to the indicated proteins; 5% of the inputs are shown (Input). Since the level of expressed Flag-NHP2 was variable after 12h of induction of this protein in cells, 2 examples of IPs are shown. **C/** WB signals were quantified as in Figure S2, significant changes are indicated: * ($p<0.05$). **D/** The expression of GFP-NHP2 protein in the HEK293 Flp-In TRex cells was induced with doxycycline 12 or 3h before observation. Double IF experiments were performed using anti-coilin (the marker of CBs) and anti-nucleolin (marker of nucleolus) antibodies. Images were acquired by a confocal microscope. GFP-NHP2 is represented in cyan, coilin in magenta, and nucleolin in yellow.

Supplemental Figure 5. Comparison of the proteins associated with GFP-GAR1, -NHP2, -SHQ1, and -NAF1. Venn diagram showing the intersection between the GFP-GAR1, -NHP2, -SHQ1, and -NAF1 proteomes defined by IP-SILAC analysis. The diagram includes all the proteins associated with a SILAC ratio up to 1.3, except ribosomal proteins.

Table S1. Excel file presenting the IP-SILAC data. Description of individual columns from the left to the right: protein name, gene name, fasta headers, number of unique peptides found in the IP, the intensity of the signal, SILAC ratio, the significance B calculated according to a method described previously by Cox and Mann (127), and the category of the protein. This significance B is equivalent to a p-value. The SILAC ratio is an enrichment value between specific IPs compared with control IPs made from cells not expressing GFP-tagged proteins. Specific IPs were made under medium (M: l-arginine [^{13}C] and l-lysine 4,4,5,5-D4) or heavy conditions (H: l-arginine [^{13}C,^{15}N] and l-lysine [^{13}C,^{15}N]), and control IP was done with light condition (L: l-arginine and l-lysine) depending on the type of lysine and arginine added in the media. The intensity of the signal is given for the condition in which the specific

IP was done (intensities M or H). The category of the associated proteins was chosen using the Gene Ontology Resource and Uniprot, as in Figure 1.

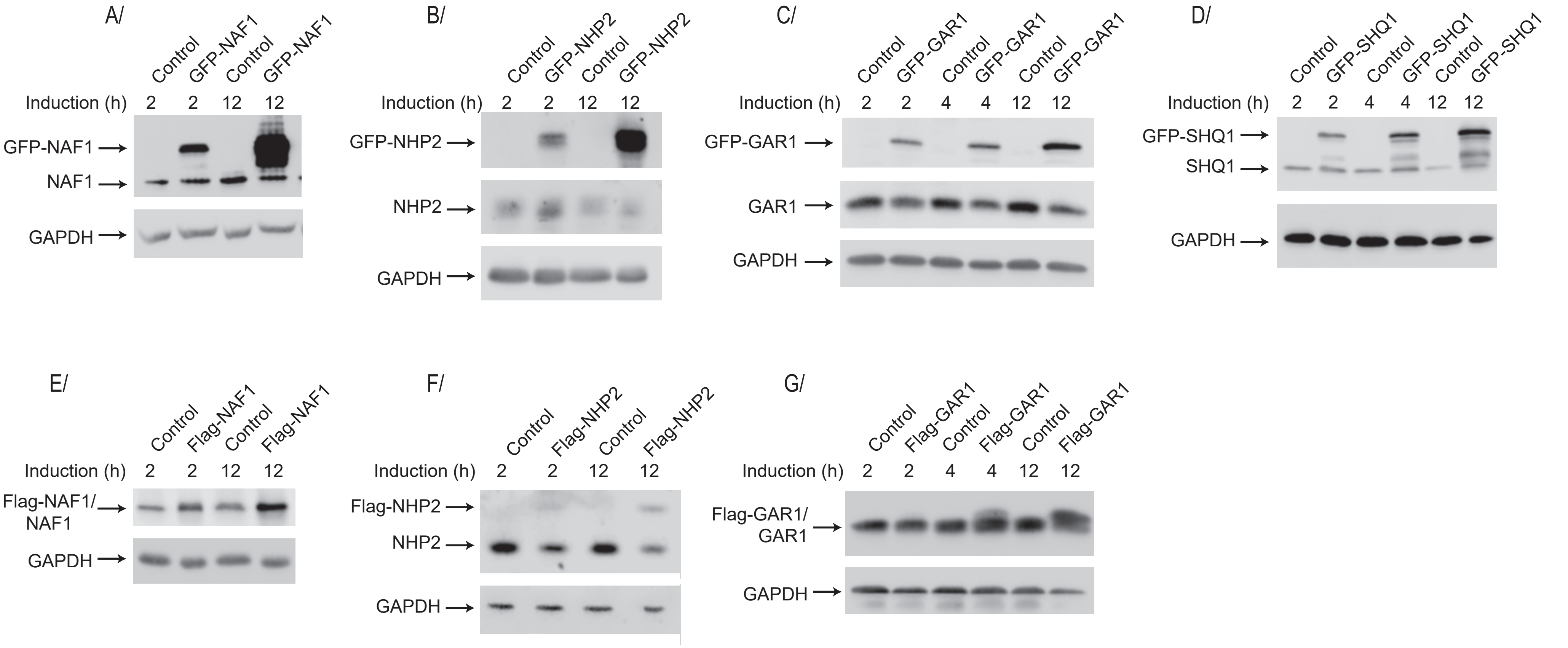

Revised Figure S1
Schlotter et al.

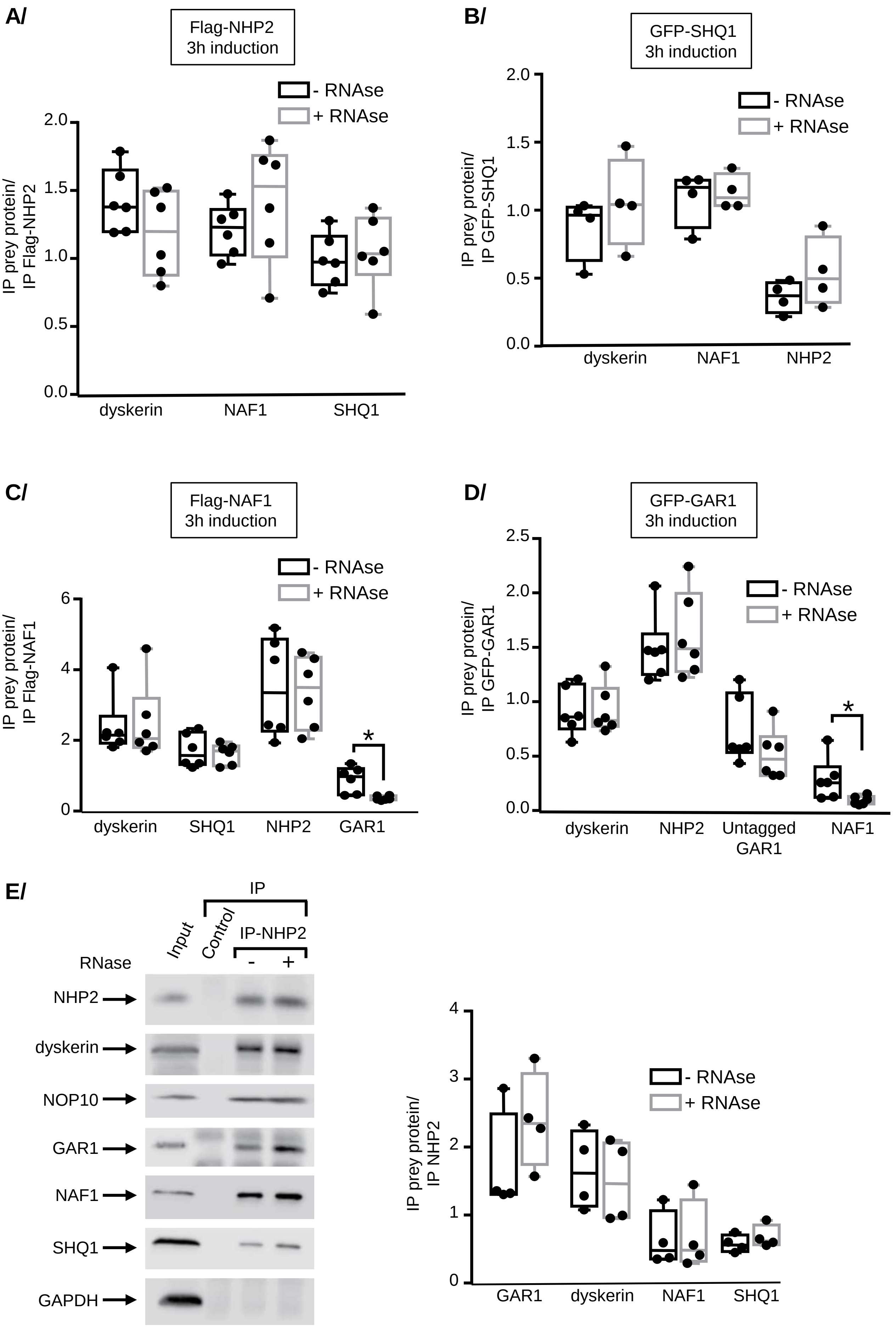

Revised Figure S2
Schlotter et al

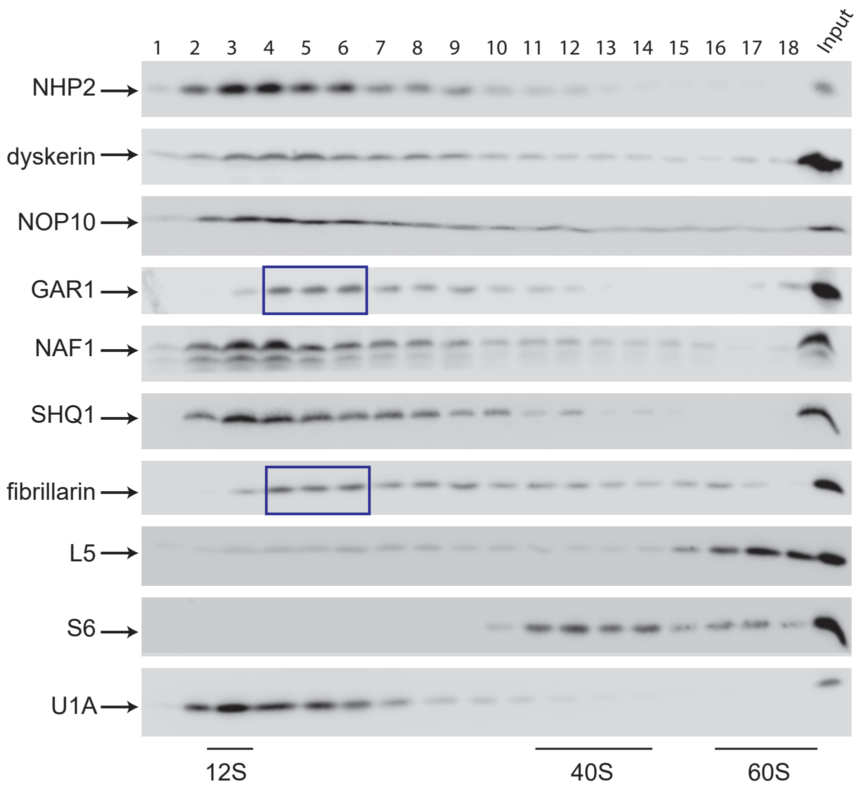

Revised Figure S3
Schlotter et al.

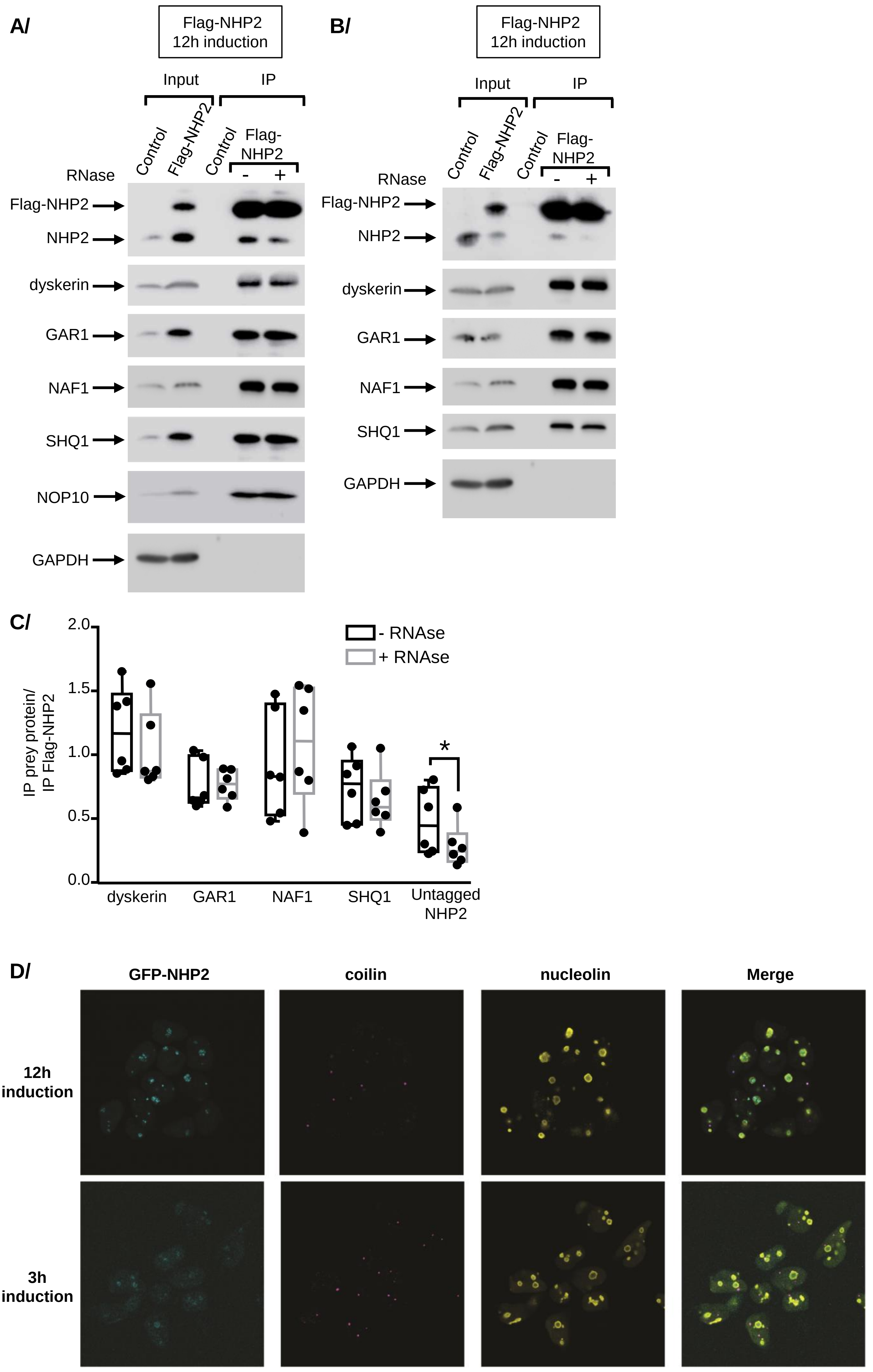

Revised Figure S4
Schlotter et al

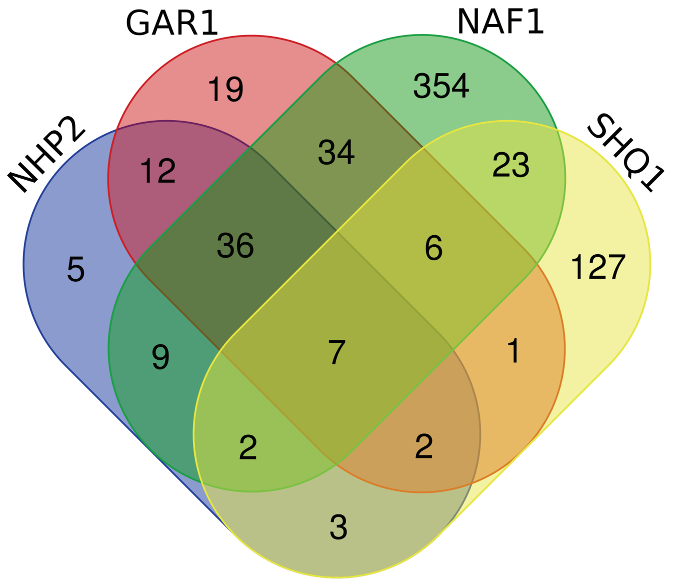

Figure S5
Schlotter et al.
